# The TATEGIRI technique: adaptation of a longitudinal aortotomy for totally endoscopic aortic valve replacement

**DOI:** 10.1186/s13019-025-03613-5

**Published:** 2025-10-17

**Authors:** Hitoki Hashiguchi, Naomi Yasuda, Akihito Ohkawa

**Affiliations:** 1 Department of Cardiovascular Surgery, Hokkaido Prefectural Kitami Hospital, Kitami, 090‑0027 Japan; 2 Department of Cardiovascular Surgery, Sapporo Medical University School of Medicine, Sapporo, Japan

**Keywords:** Totally endoscopic aortic valve replacement, Minimally invasive cardiac surgery, Longitudinal aortotomy, TATEGIRI, Surgical exposure

## Abstract

**Background:**

Endoscopic aortic valve replacement (TE‑AVR) is hindered by restricted visualization and instrument maneuverability. We describe TATEGIRI (Japanese: “vertical cut”), a longitudinal aortotomy adapted for the endoscopic setting.

**Methods:**

Between August 2023 and August 2024, 28 consecutive patients (23 sternotomies and five MICS) underwent AVR using the TATEGIRIapproach. Perioperative variables, procedural feasibility, and early outcomes were prospectively assessed.

**Results:**

The median cardiopulmonary bypass time was 142 min and the aortic cross-clamp time was 97 min. Valve size distribution was 19 mm in one patient (3.6%), 23 mm in eight (28.6%), 25 mm in seven (25%), 27 mm in five (17.9%), and 29 mm in one (3.6%). The mean prosthesis diameter was 24.5 ± 3.1 mm. In the five totally endoscopic cases, the median prosthesis diameter was 25 mm (interquartile range [IQR] 23–27) versus 23 mm (IQR 23–25) in full‑sternotomy cases (*p* = 0.09). Two patients underwent reintervention (one reexploration for bleeding and one sternal rewiring). There were two 30‑day mortalities (sepsis and stroke, both in patients who underwent sternotomy). Paravalvular leakage was not observed. The median length of the hospital stay was 13 days. Follow-up CT at a median of 3.5 months showed no aneurysmal change or stenosis and demonstrated an average 2 mm reduction in aortic diameter along the aortotomy line.

**Conclusions:**

TATEGIRI longitudinal aortotomy provides consistent three-quarters (~ 270°) annular exposure while preserving a straight, fully visible suture line. Early results demonstrated technical feasibility and favorable short-term outcomes, supporting wider adoption and evaluation in comparative studies.

**Supplementary Information:**

The online version contains supplementary material available at 10.1186/s13019-025-03613-5.

## Introduction

Longitudinal aortotomy has long been used under median sternotomy for annular enlargement; however, its systematic application in a totally endoscopic environment is scarce. Tokoro et al. first reported a three-port totally endoscopic aortic valve replacement (AVR) with clinical results equivalent to those of transaxillary mini-thoracotomy and a median hospital stay of 10 days [1]. Subsequently, the same group confirmed the feasibility of the anterolateral approach by using standard prostheses in 30 patients [2]. Furthermore, Vola et al. described an anatomical rationale for endoscopic exposure. However, they continued to rely on short transverse aortotomies and reported excellent valve visibility at the expense of curved suture lines [3]. Moscarelli et al. reviewed robotic totally endoscopic coronary artery bypass and highlighted how oblique aortotomies generate redundant flaps that complicate knot-tying in the endoscopic field [4].

More recently, Gu et al. published a series of 31 cases of totally endoscopic AVR in China using an oblique incision and underscored the ongoing need for enhanced annular exposure and straight‑line closure under endoscopic vision [5]. European and Southeast Asian data are also emerging; Cresce et al. reported the operative results of minimally invasive endoscopic AVR and Nguyen et al. presented the early outcomes of totally 3D endoscopic AVR from a single Vietnamese center [6, 7].

## Patients and methods

### Study design

Single‑center prospective cohort; the Institutional Review Board approval was waived for this anonymized observational analysis.

### Patient population

Twenty-eight consecutive patients who underwent isolated AVR between August 2023 and August 2024 were included in this study (Table 1). Patients who underwent concomitant aortic replacement were excluded.

### Operative technique

Detailed port positions and instrumentation are provided in Supplementary Video 1. The key steps are summarized as follows.

**Fig. 1:**
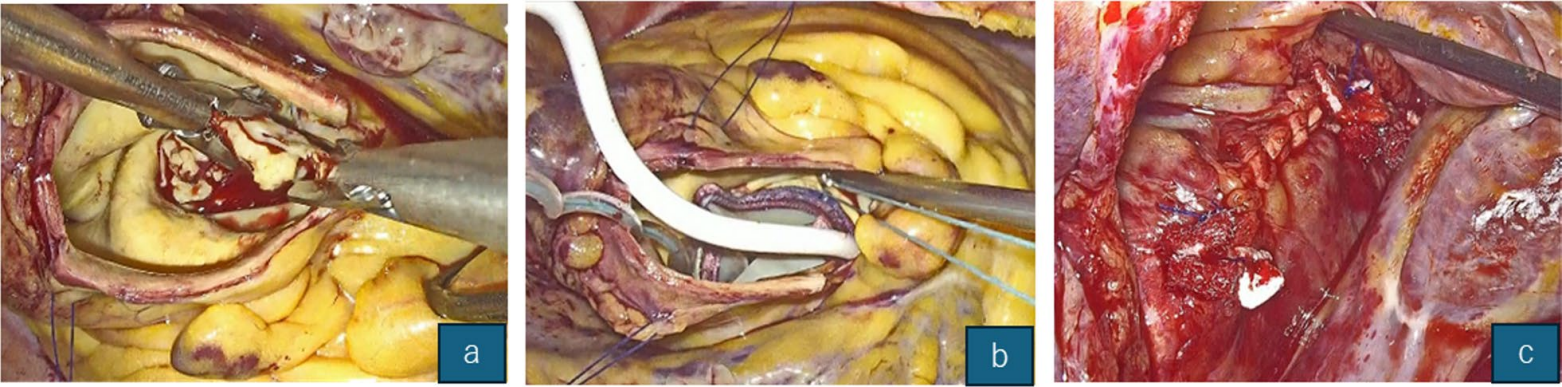
Total endoscopic view of the TATEGIRI incision. (**a**) Initial puncture site and longitudinal extension toward the non-coronary sinus. (**b**) Complete 270° annular exposure after leaflet excision. (**c**) The suture line remained linear and entirely visible within a single endoscopic field

- Incision direction (illustrated in Fig. 1a–c): After withdrawal of the antegrade cardioplegia delivery needle, the puncture site on the anterior ascending aorta was extended distally with a longitudinal incision toward the non-coronary sinus of Valsalva (NCC nadir) and routinely stopped approximately 1 cm proximal to the sinotubular junction (STJ). In patients with STJ diameters equal to or less than the left ventricular outflow tract (LVOT), we extended the incision across the STJ and reconstructed the wall with a boat-shaped Dacron patch to keep sinus geometry.
- Valve implantation: A standard horizontal mattress and interrupted suture or rapid‑deployment Edwards INTUITY were used according to the annular size; no annular‑enlargement procedure was required.
- Closure: Two‑layer running closure under direct endoscopic vision.

## Results

### Demographics and operative data

Table 1 lists baseline characteristics; Table 2 summarises operative metrics.

### Early outcomes

The median postoperative length of hospital stay was 13 days (interquartile range [IQR] 9–16 days). Two patients (7.1%) died within 30 days: one from a catheter-related bloodstream infection with sepsis and one from cardioembolic stroke during postoperative atrial fibrillation; both occurred in sternotomy cases. One patient (3.6%) required reexploration for bleeding, and two (7.1%) underwent sternal rewiring for sternal dehiscence. A permanent pacemaker was implanted in one patient (3.6%) for complete atrioventricular block. No paravalvular leak was detected on predischarge echocardiography. Early follow-up CT (median 3.5 months) revealed no aneurysmal changes and a mean 2 mm decrease in the aortic diameter at the incision site.

## Discussion

Our data suggest that longitudinal aortotomy offers three endoscopy-specific advantages over oblique/transverse techniques: (1) linear exposure provides up to three‑quarters (~ 270°) annular visibility and eliminates blind corners; (2) because the longitudinal continuity of the aortic wall is preserved, the annulus remains stable and a valve sizer can be inserted *before* any annular sutures are placed, permitting accurate sizing and minimizing malalignment; and (3) the straight suture line remains entirely in the camera view, expediting closure. These features translated into median cross-clamp times close to those reported in the contemporary literature for mini-sternotomy AVR (55–70 min) [3, 6, 7].

Limitations include (i) single‑center design; (ii) predominance of full sternotomy—only five of the first 28 cases were performed through a totally endoscopic or mini‑thoracotomy route; and (iii) absence of a parallel control group. After completing this exploratory series, we continued to endoscopically apply the technique and collected 16 consecutive totally endoscopic aortic valve replacement (TE‑AVR) cases at our institution. Outcomes from the enlarged minimally invasive cohort will be analyzed and reported in a dedicated follow-up study. In addition, a matched comparison between transverse and oblique TE-AVR is currently underway.

## Conclusions

The TATEGIRI technique appears to be reproducible and provides consistent exposure during TE‑AVR without additional morbidity. Broader adoption depends on multicenter verification.

**Table 1:** Patient demographics (TATEGIRI *n* = 28)

Variable	Value
Age, years, median (IQR)	81 (71–85)
Male sex, n (%)	19 (68)
Body‑surface area, m², mean ± SD	1.59 ± 0.18
STS‑PROM, %, mean ± SD	3.2 ± 1.1
Aortic stenosis, n (%)	23 (82)
Aortic regurgitation, n (%)	5 (18)
Hypertension, n (%)	14 (50)
Diabetes mellitus, n (%)	6 (21)
Chronic kidney disease (> Stage 3), n (%)	4 (14)

**Table 2:** Operative and postoperative outcomes (*n* = 28)

Variable	Median (IQR) or *n* (%)
Cardiopulmonary bypass time (min)	142 (100–200)
Aortic cross-clamp time (min)	97 (65–128)
Postoperative length of stay (days)	13 (9–16)
Re-exploration for bleeding	1 (3.6%)
Sternal rewiring	2 (7.1%)
Permanent pacemaker implantation	1 (3.6%)
Paravalvular leak	0 (0%)
30-day mortality	2 (7.1%)

## Supplementary Information

Supplementary Material 1

## Data Availability

No datasets were generated or analysed during the current study. De‑identified datasets are available from the corresponding author upon reasonable request.
